# Evaluation of Antidiarrheal Activity of 80% Methanolic Extract of the Leaves of *Cordia africana* (Lamiaceae) in Mice

**DOI:** 10.1155/2021/3627878

**Published:** 2021-11-27

**Authors:** Yared Andargie Ferede, Woretaw Sisay Zewdu, Mulugeta Molla Zeleke, Muluken Adela Alemu

**Affiliations:** School of Pharmacy, College of Medicine and Health Science, Debre Tabor University, Debre Tabor, Ethiopia

## Abstract

**Background:**

Diarrheal disease is a major cause of morbidity and mortality throughout the world, particularly in developing countries. Currently available drugs are linked with adverse effects, contraindications, and risk of resistance. Traditionally, the leaf concoction of *Cordia africana* is claimed to be used for diarrhea. However, the safety and efficacy of the leaf extract have not been scientifically approved yet. Therefore, the study was conducted to validate its antidiarrheal activity and safety profile in mice.

**Method:**

The hydromethanolic extract was obtained by the cold maceration technique in 80% methanol. Phytochemical screening tests were done for secondary metabolites by using standard tests. The antidiarrheal activity of the test extract at the doses of 100, 200, and 400 mg/kg was evaluated by using castor oil-induced diarrheal, gastrointestinal transit, and enteropooling models in mice.

**Result:**

In an acute toxicity study, there were no visible signs of toxicity and mortality following a single oral administration of 2000 mg/kg. Phytochemical screening tests revealed the presence of alkaloids, saponins, flavonoids, terpenoids, phenols, and tannins. The hydromethanolic extract significantly prolonged the onset of diarrhea and reduced the weight of wet and total feces at 100 (*P* < 0.01), 200 (*P* < 0.001), and 400 mg/kg (*P* < 0.001) in the castor oil-induced diarrheal model. However, in the gastrointestinal transit model, a significant (*P* < 0.001) reduction in the charcoal meal travel was observed in the middle (200 mg/kg) and higher (400 mg/kg) test doses. Similarly, the extract produced a significant (*P* < 0.001) reduction in the weight and volume of intestinal contents at the aforementioned doses.

**Conclusion:**

The study demonstrated that the test extract showed promising antidiarrheal activity. Hence, this study supports its antidiarrheal use in Ethiopian folklore medicine.

## 1. Introduction

Diarrhea is a gastrointestinal disorder which is characterized by an alteration in a normal bowel movement and an increase in the water content, volume, and weight or frequency of stool evacuation three or more times per day [1]. It is a major cause of morbidity and mortality throughout the world, particularly in developing countries. Studies done in 2021 reported that diarrhea remains the second leading cause of death among children under five years of age, globally, next to pneumonia [2]. Of all child deaths from diarrhea, 78% occur in African and Southeast Asian regions. In Ethiopia, its mortality rate is the 5th highest in the world, next to India, Nigeria, Pakistan, and the Democratic Republic of Congo [3]. Moreover, diarrhea remained as one of the top 10 causes of death in Ethiopia [4].

Medicinal plants play an invaluable role in the development of potent therapeutic agents. Currently, it is estimated that about 80% of people in developing countries still rely on traditional medicine for their primary health care [5]. They are currently in demand, and their popularity is increasing over time as a potential source of modern medicine. Approximately 25% of modern medicines are directly descended from natural products. Many others are synthetic analogues built from prototype compounds isolated from medicinal plants [6, 7].

There are a wide range of medicinal plants that play an important role in treating and managing diarrhea. Among these, the bark extract of *Albizia gummifera*; the leaf extract of *Calpurnia aurea*, *Cordia africana* (Lam.), *Artemisia afra*, and *Croton macrostachyus*; the seed extract of *Coffea arabica*; and the root extract of *Echinops kebercho*, *Ensete ventricosum*, *Cucumis ficifolius*, *Leonotis ocymifolia*, and *Caylusea abyssinica* have been widely used for the treatment of diarrhea and related gastrointestinal disorders in Ethiopian folklore medicine [8–11]. However, the therapeutic potential and safety profile of some of these medicinal plants such as *Cordia africana* (Lam.) have not been scientifically validated yet.

*Cordia* is a pantropical genus of flowering plants that belongs to the Boraginaceae family. The plant family comprises about 100 genera and more than 2000 species [12], of which *Cordia africana* (Lam.) is one of the most common forest tree species in the family. Locally, it is known as wanza and is commonly named as East African Cordia or large-leafed Cord17a or Sudan teak in English [13]. It is found in the moist evergreen highlands and riverine forests of the northwest and southwest highlands and is very common in western Ethiopia between 550 and 2600 m where the rainfall range is 700 to 2000 mm per year [14].

In an ethnobotanical survey conducted in northwest Ethiopia, different parts of *Cordia africana* have been reported to be used for various ailments [15] such as liver disease, amebiasis, stomachache, and particularly, the leaf part squeezed with a drop of water and drunk the concoction with a cup of coffee is claimed to be used for diarrhea in the Afar and Tigray regions of Ethiopia [14, 16].

There are various experimental studies which show various parts of the plant are proved to have various activities including *in vivo* anti-inflammatory, antioxidant, and anti-nociceptive activities of ethanolic extracts of the plant [13, 17–19], anti-inflammatory, antibacterial, and antioxidant activities of the leaf and stem bark extracts [20], and antidiarrheal activity of the root bark extract [15].

Currently available drugs are linked with adverse effects and contraindications [21]. Drug resistance is also another challenge to think about antibiotics used in the treatment of diarrhea [22]. Though the leaf extract of *Cordia africana* (Lam.) has been claimed to be used as an antidiarrheal agent, its therapeutic potential and safety profile have not been scientifically approved yet. Therefore, the study was conducted to validate its antidiarrheal activity and safety profile. The result of this study will also be a basis for further research for structural elucidation, for studying the structural activity relationship and the mechanism of action of the active biological compound.

## 2. Materials and Methods

### 2.1. Drugs, Chemicals, and Materials

Absolute methanol (Carlo Erba reagents, S.A.S, France), castor oil (Amman Pharmaceutical Industries, Jordan), activated charcoal (Acuro Organics Ltd, New Delhi, India), loperamide (Daehwa Pharmaceutical, Republic of Korea), distilled water (Ethiopian Pharmaceutical Manufacturing Factory, Epharm, Ethiopia), Wagner's reagent (Research-Lab Fine Chem Industries, India), lead acetate 1% (Guangdong Guanghua chemicals factories, China), sodium hydroxide (Blulux Laboratories (P) Ltd., India), ferric chloride (Blulux Laboratories (P) Ltd., India), chloroform (Hi-Media Laboratory Reagents, India), sulphuric acid (HiMedia Laboratories Pvt. Ltd., India), and lyophilizer (Lab freeze Instruments Group Co., Ltd., Japan) were used. All experimental supplies were analytical grade and purchased from Gondar Pharmaceutical Fund Supply Agency and nearby suppliers.

### 2.2. Experimental Animals

Healthy Swiss albino mice of either sex, weighing 25–30 gm, and aged 6–8 weeks were used to conduct the study. The mice were obtained from the Ethiopian Public Health Institute (EPHI). They were housed in plastic cages with softwood shavings and chips as bedding at room temperature (25 ± 2°C) and were kept under a 12/12 hour light to dark cycle. They were allowed free access to standard pellet feed and water ad libitum and were acclimatized to the working environment for one week before commencing the actual experiment. The animals were deprived of food for 18 hours before starting the experiment. The study was carried out according to the National Research Council Guide for the Care and Use of Laboratory Animals and Organization for Economic Co-operation and Development (OECD) guidelines [23].

### 2.3. Plant Material Collection and Identification

The fresh leaves of *Cordia africana* (Lam.) were collected from Debre Tabor town, which is 655 km away from the capital city of Ethiopia, Addis Ababa, in the northwest direction and 99 km away from Bahir Dar town, the city of the Amhara region, in February, 2021. In the meantime, the fresh leaves of the plant were brought and sent to Debre Tabor University for authentication, and the plant specimen was deposited there in the Department of Biology, Natural Science Faculty, Debre Tabor University with voucher no. (AY10) for future reference. After authentication, the leaves of *Cordia africana* (Lam.) were collected in bulk, washed gently with tap water, and dried at room temperature under shade for 2 weeks. Then, the dried leaves were reduced to coarse powder by using a mortar and pestle and kept in plastic container until use for extraction.

### 2.4. Preparation of the Crude Extract

A total of 600 gm of coarsely powdered leaves of *Cordia africana* was macerated with 2000 ml of 80% methanol as a solvent in an Erlenmeyer conical flask for 72 hours by occasional hand shaking. On the third day, after exhaustive extraction, the filtrate was first separated from the marc by using a muslin cloth, and then, further filtered by Whatman filter paper (No. 1). After filtration, the supernatant was collected and the remaining residue was remacerated twice for a total of six days with 2500 ml of a fresh solvent of 80% methanol to maximize the percentage yield. The marc was pressed, and the filtrates from each extraction were combined together and evaporated using a hot oven adjusted at 40°C to remove methanol. Then, the concentrated aqueous solution filtrate was placed in a deep freezer overnight followed by drying with a lyophilizer set at −50°C to remove water. Finally, the percentage yield of the crude extract was calculated and kept in an airtight container at −20°C until used for the intended experiment [13].

### 2.5. Phytochemical Screening Test

The hydromethanolic extract of the leaf of *Cordia africana* (Lam.) was screened for the presence of active phytochemical constituents by using standard tests [24]. Thus, tests for alkaloids, saponins, flavonoids, terpenoids, phenols, steroids, glycosides, and tannins were performed.

### 2.6. Acute Toxicity Test

An acute toxicity test was conducted according to OECD 425 (2008) guidelines as described below. Five female, nonpregnant, and adult Swiss albino mice weighing 25–30 gm were randomly grouped and kept in a plastic cage. After being fasted for food for 3 hours, a single female mouse was treated with a single dose of 2000 mg/kg of the crude extract by using oral gavage. Then, the mouse was strictly observed every 30 minutes for 4 hours for any signs of toxicity and mortality within the first 24 hours. Based on the outcome of the first animal, the remaining 4 female mice were fasted for 3 hours and then treated sequentially once with a similar dose of the test extract. Then, the animals were observed for any signs of toxicity and mortality for 14 days [25].

### 2.7. Grouping of Animals and Dosing

For all three models, thirty Swiss albino mice of either sex (25–30 gm) were randomly allocated into five groups, each having six. The negative control was assigned as group I and given a vehicle (distilled water, 10 mg/kg). Test groups II, III, and IV were given the test extract at a dose of 100 mg/kg (CA100), 200 mg/kg (CA200), and 400 mg/kg (CA400), respectively, whereas the fifth group (group V) was assigned as a positive control and the standard drug loperamide 3 mg/kg was given in all three diarrheal models. The given doses of the respective treatments were given once, and the doses of the test extract were determined based on an acute toxicity study.

### 2.8. Determination of Antidiarrheal Activity

#### 2.8.1. Castor Oil-Induced Diarrhea Model

The method followed by Sisay et al. and Abdela was utilized to conduct the study with slight modification [26, 27]. Swiss albino mice of either sex were deprived of food for 18 hours with free access to water and randomly divided into five groups, each having six, and treated as described under the grouping and dosing section. All treatments were delivered via the oral route. After one hour, diarrhea was induced by the administration of 0.5 ml of castor oil (CO) orally to each mouse. The animals were then housed individually in a separate transparent cage, in which the floor was lined with white clean paper. Thereafter, they were strictly observed for 4 hours, and then, the onset of diarrhea, frequency of defecation, and weight of fecal output were recorded. The percentages of diarrheal inhibition and weight of fecal output were calculated by using the following formula:

(1) $$\begin{array}{c} \text{\% of inhibition }=\;\frac{\text{mean number of WFC}-\text{mean number of WFT}}{\text{mean number of WFC}}\times 100\text{,} \end{array}$$

where WFC = wet feces in the control and WFT = wet feces in the test group.

(2) $$\begin{array}{c} \text{Percentage of wet fecal output}=\frac{\text{mean weight of wet feces of each group}}{\text{mean weight of wet feces of each group}}\times 100\text{,}\\ \text{Percentage of total fecal output}=\frac{\text{mean weight of total feces of each group}}{\text{mean weight of total feces of control}}\times 100\text{.} \end{array}$$

#### 2.8.2. Castor Oil-Induced Gastrointestinal Motility Test

All mice were fasted for 18 hours with free access to water and divided into five groups, each having six, and treated as described earlier. An hour later, each mouse was given 0.5 ml of CO orally. Then, after 1 hour, all mice received 1 ml of 5% activated charcoal suspension. The animals were then sacrificed after 30 minutes, and the small intestine was dissected out from the pylorus to cecum. The distance travelled by the charcoal meal and the total length of the intestine were then measured. The peristaltic index and percentage of inhibition were then expressed using the formula as follows [28]:

(3) $$\begin{array}{c} \text{peristalsis index}=\frac{\text{distance travelled by charcoal meal}}{\text{length of small intestine}}\times 100,\\ \text{percentage of inhibition}=\frac{\left(Dc-Dt\right)}{Dc}\times 100, \end{array}$$

where Dc is the mean distance travelled by the control and Dt is the mean distance travelled by the test group.

#### 2.8.3. Castor Oil-Induced Enteropooling Activity

Intestinal fluid accumulation was determined using the method described by Adeyemi and Akindele [29]. Mice of either sex were fasted for 18 hours and treated as described earlier an hour before administering CO. Then, the mice were sacrificed by cervical dislocation after 1 hour of being induced with castor oil. The abdomen of each mouse was then opened, and the small intestine was removed and weighed. The intestinal contents were collected, and the volume was measured. The small intestine was reweighed, and the difference between full and empty intestine was calculated. Finally, the percentage inhibition of the volume and weight of intestinal contents were determined by using the following formula [30]:

(4) $$\begin{array}{c} \text{percentage of inhibition}=\frac{\left(\text{MVICC}-\text{MVICT}\right)}{\text{MVICC}}\times 100, \end{array}$$

where MVICC = mean volume of the intestinal content of the control group and MVICT = mean volume of the intestinal content of the test group.

(5) $$\begin{array}{c} \text{Percentage of inhibition}=\frac{\left(\text{MWICC}-\text{MWICT}\right)}{\text{MWICC}}\times 100, \end{array}$$

where MWICC is the mean weight of the intestinal content of the control group and MWICT is the mean weight of the intestinal content of the test group.

### 2.9. In Vivo Antidiarrheal Index

The *in vivo* antidiarrheal index (ADI) of the positive control and the tested group was determined by combining three parameters taken from the aforementioned models using the following formula developed by Aye-Than et al. [28]:

(6) $$\begin{array}{c} \text{in vivo anti}-\text{diarrheal index }=\sqrt[{3}]{\text{Dfreq}\times \text{meq}\times \text{Pfreq}}, \end{array}$$

where Dfreq = delay in defecation time or diarrheal onset (in % of control), Gmeq = gut meal travel reduction (in % of control), and Pfreq = purging frequency as the number of wet stool reduction (in % of control).

### 2.10. Data Quality Control

The data quality was maintained by grouping experimental animals by simple random sampling technique, blinding the data collection system of all parameters, maintaining and applying standard procedures, and using analytically graded materials.

### 2.11. Statistical Analysis

Data were entered and analyzed using SPSS version 20. The experimental results were expressed as mean ± standard error of the mean (SEM), and statistical significance was carried out by employing one-way analysis of variance (ANOVA) followed by a Tukey post hoc test for multiple comparisons. The results were considered statistically significant when the *P* value is less than 0.05.

## 3. Results

### 3.1. Extraction Yields of Plant Material

The percentage yield of the crude extract was found to be 14% (w/w) with an actual mass of 84 gm out of 600 gm of coarse powder of *Cordia africana.*

### 3.2. Phytochemical Screening Test

The preliminary phytochemical screening of the hydromethanolic extract of the leaves of *Cordia africana* revealed the presence of alkaloids, saponins, tannins, phenols, terpenoids, and flavonoids (Table 1).

### 3.3. Acute Oral Toxicity Study

The hydromethanolic extract of the leaves of *Cordia africana* produced neither overt toxicity nor death during 14 days of follow-up following oral administration of a single dose of 2000 mg/kg of the extract. Besides, the toxicity study did not show any signs and symptoms of toxicity, such as behavioral, neurological, autonomic, or physical changes.

### 3.4. Effects of the Crude Extract on the Castor Oil-Induced Diarrheal Model

In this model, the hydromethanolic leaf extract of *Cordia africana* significantly (*P* < 0.05) prolonged the onset of diarrhea at all test doses of the test extract with a better effect exhibited at the highest dose (*P* < 0.001) as compared to the negative control. In the same fashion, the test extract at the dose of 200 and 400 mg/kg also decreased highly significantly the average weight of wet feces (*P* < 0.001) and total fecal output relative to the control group. Whereas, the plant extract at the dose of 100 mg/kg reduces the aforementioned parameters less significantly (*P* < 0.01). In addition, the finding showed that the percentage of diarrheal inhibitions were 11%, 76.7%, and 83.6% at test doses of 100, 200, and 400 mg/kg, respectively (Table 2).

### 3.5. Effects of the Crude Extract on Castor Oil-Induced Intestinal Transit in Mice

In the negative control group, the distance travelled by the charcoal meal was found to be 44.5 ± 0.76, and its peristaltic index was 87.3 ± 1.51. The test extract was able to significantly reduce the distance travelled by the charcoal meal at doses of 200 mg/kg (23.3 ± 0.49, *P* < 0.001) and 400 mg/kg (13.0 ± 0.97, *P* < 0.001). In contrast, at a dose of 100 mg/kg, the test extract did not show a statistically significant reduction of the propulsion of the charcoal meal along the intestine as compared with the negative control. In this model, the test extract at a dose of 400 mg/kg exhibited a significant reduction in the distance travelled by the charcoal meal (70.8%), which is comparable with the standard drug loperamide (71.5%) (Table 3).

### 3.6. Effects of the Crude Extract on Castor Oil-Induced Enteropooling

The volume and weight of the intestinal contents of the negative control were 1.30 ± 0.10 and 1.50 ± 0.09, respectively. The plant extracts were able to significantly inhibit castor oil-induced gastrointestinal fluid accumulation at the test doses of 200 and 400 mg/kg. However, the activity of the test extract at the dose of 100 mg/kg was not statistically significant. Hence, the volume of intestinal contents for extract treated groups at the doses of 200 and 400 mg/kg were 0.75 ± 0.08 (*P* < 0.01) and 0.50 ± 0.10 (*P* < 0.001), respectively. Similarly, the test extract significantly decreased the weight of intestinal contents at 200 mg/kg (0.85 ± 0.08, *P* < 0.001) and 400 mg/kg (0.73 ± 0.15, *P* < 0.001) relative to the negative control. In this model, the percent inhibition in the weight of intestinal content was found to be 43.3% and 51.3% at the doses of 200 and 400 mg/kg, respectively. The maximal effect in reduction of the aforementioned parameter was observed at the highest dose of the extract (51.3%) which is comparable to the standard drug (53.3%), as shown below (Table 4).

### 3.7. Effect of the Crude Extract on In Vivo Antidiarrheal Index

The *in vivo* antidiarrheal index (ADI) of the test extract was determined by combining three parameters, as shown in Table 5. These are the delay in diarrheal onset (time of onset, Dfreq), gut meal travel distance reduction (Gmeq), and purging frequency in the number of wet stools (Pfreq). The ADI values were 12.2, 69.9, and 89.8 at doses of 100, 200, and 400 mg/kg, respectively. These findings demonstrated that the plant extract exhibited dose-dependent antidiarrheal indices with the maximum effect observed at 400 mg/kg of the test extract.

## 4. Discussion

Human beings commonly use different parts of medicinal plants as remedies for various ailments, including diarrheal disease, without any scientific evidence about their safety and efficacy [27]. It is, therefore, important to properly evaluate the safety and efficacy profiles of medicinal plants that are being used in traditional medicine. Several studies have scientifically approved traditionally used antidiarrheal plants by evaluating the effects of these plants on gastrointestinal motility, water, and electrolyte secretion using animal models [31]. The need for newer, more effective, cheaper, and safer antidiarrheal drugs has become a paramount concern to have safe and cost-effective therapeutic alternatives [26]. Hence, the study was proposed and conducted to validate the claimed antidiarrheal effect of *Cordia africana* leaves using three antidiarrheal experimental models.

The acute toxicity study revealed that the plant extract was found to be safe as no sign of overt toxicity was observed at the limit test dose of 2000 mg/kg in mice. At this dose, mortality and delayed toxicity were not observed in the 14 -day follow-up period. The result indicated that the LD50 value of the test extract is estimated to be above 2000 mg/kg. Overall, the finding demonstrated that the leaf of the plant extract is tolerable and safe following oral administration which validates the safe use of the plant in traditional settings.

The antidiarrheal activity of the 80% methanol extract of *Cordia africana* leaves was investigated for its activity against diarrheal disease in all three models using castor oil as the diarrhea-inducing agent. There are several mechanisms proposed to explain the diarrheal effect of castor oil: production of an irritant laxative effect mediated by its active metabolite ricinoleic acid released by intestinal lipases. Ricinoleic acid produces local irritation and inflammation of the intestinal mucosa, causing the release of prostaglandins that eventually increase gastrointestinal motility, net secretion of water and electrolytes [32]; inhibition of intestinal Na+/K + -ATPase activity, thus reducing normal fluid absorption [33]; and activation of adenylate cyclase or mucosal cAMP-mediated active secretion [34] and nitric oxide [35]. Therefore, castor oil was used as an inducing agent in all three models as it produces diarrhea in the same fashion as the pathophysiologic processes. Loperamide is used as the standard drug because it antagonizes the action of castor oil efficiently due to its antimotility and antisecretory properties. Hence, currently, it is one of the most efficacious and widely employed antidiarrheal agents [36].

The findings of the present study revealed that the hydromethanolic extract of the leaves of *Cordia africana* was found to be effective against castor oil-induced diarrhea in different antidiarrheal models. The first model being castor oil-induced diarrheal model was designed to assess the overall antidiarrheal activities of the extract. In this model, the onset of defecation, the frequency, and weight of wet feces, more importantly wet feces, were determined in the 4 hour follow-up period. Hence, agents which inhibit the number and weight of feces are considered to have antidiarrheal activity [37].

In the aforementioned parameters, the test extract exhibited statistically significant activity at all test doses. The percent reduction was increased with a corresponding increase in the dose of the test extract. This increasing activity of the hydromethanolic extract with increasing the dose speculated that the extract inhibits diarrhea more effectively at relatively higher dose [21]. This finding is in concordance with other reports of other species of plants in which extracts of plants have shown to exert antidiarrheal effect at higher doses [38]. Furthermore, these results are also in line with the findings of the previous study conducted on the root bark of *Cordia africana* [15]. This could imply that the bioactive constituents of the extract, which are responsible for antidiarrheal activities, are more likely to be concentrated at higher doses. The significant reduction in all parameters showed that the hydromethanolic extract of the leaves of *Cordia africana* is effective as an antidiarrheal agent. Since castor oil causes diarrhea by inhibiting fluid and electrolyte absorption and thus resulting in intestinal peristalsis [39], one possible mechanism for its antidiarrheal activity could be the test extract's ability to enhance fluid and electrolytic absorption through the gastrointestinal tract.

Previous studies have been shown that nonsteroidal anti-inflammatory drugs (NSAIDs) could inhibit castor oil-induced diarrhea [40]. Interestingly, there are studies that show the leaves of the plant have anti-inflammatory activity [20]. Hence, the antidiarrheal action exerted by the extract might be associated with the inhibition of prostaglandin formation. This suggestion is validated by the fact that castor oil-induced diarrhea is related to stimulation of prostaglandin biosynthesis [30]. Furthermore, the antidiarrheal activity might be from its inhibitory activity on nitric oxide and platelet-activating factors production, as there is confirmed evidence in which nitric oxide syntheses and platelet activating factor inhibitors are known to delay castor oil-induced diarrhea [41, 42].

The phytochemical analysis of the extract revealed the presence of various phytochemical constituents. Among these, identified flavonoids are known to inhibit the biosynthesis of cyclooxygenase 1 and 2 (COX-1 and COX-2), thereby inhibiting prostaglandin production [43]. In addition, tannins are known to reduce peristaltic movement and intestinal secretion by forming protein tannates in the intestinal mucosa [44]. All the aforementioned secondary metabolites were screened from the leaves of the plant. Therefore, the antidiarrheal activity of *C. africana* leaf extract observed in this study might be attributed to these secondary metabolites.

Mostly, antidiarrheal agents produce their effect by decreasing secretion and/or reducing the peristaltic movement of the GI smooth muscles [45]. Therefore, to get further information about the mechanism of its antidiarrheal activity, the extract was tested by using enteropooling and motility tests.

The charcoal meal test in mice is a method used to study the effect of drugs on the motility of the intestine. In this model, the test extract of *C. africana* at the doses of 200 and 400 mg/kg produced a statistically significant reduction in gastrointestinal transit in a dose-dependent manner, especially the effect of the maximum dose being closer to the effect of loperamide. However, the effect of the extract at 100 mg/kg was found to be insignificant. This finding suggests that the extract at middle and higher doses is capable of inhibiting the peristaltic movement of the intestine, thereby indicating the presence of antimotility activity.

The antimotility effect of the plant extract may be by mechanisms that involve the inhibition of serotonergic and cholinergic activity. It has been reported that the activation of serotonergic receptors (5-HT1, 5-HT2, 5-HT3, and 5-HT4 receptors) by endogenous serotonin causes gut motility and affects bowl transit in experimental animals [46]. Hence, antiserotonin action can be one possible mechanism by which the plant extract reduced intestinal motility. The standard antidiarrheal drug loperamide used for the positive control is a synthetic opiate agonist activating the *μ*-opioid receptors, thereby inhibiting the release of acetylcholine and, subsequently, relaxing smooth muscle tone inside the intestine wall, as shown in the previous study. These physiological processes lead to inhibition of peristalsis, hence increasing intestinal transit time [47]. Thus, the antidiarrheal activity of the test extract due to intestinal antimotility could also be possible by inhibiting the release of acetylcholine.

Phytochemical constituents such as flavonoids, terpenoids, and tannins are reported to possess antidiarrheal activity due to their ability to inhibit intestinal motility. Hence, the significant antimotility effect of the extract may be related to the synergistic inhibitory effect of flavonoids and tannins on castor oil-induced gastrointestinal motility [44, 48, 49].

The third model, enteropooling model, was conducted to assess the secretary components of diarrhea. In this model, similar to the findings in the gastrointestinal motility test, the test extract showed a significant reduction in both the mean weight and the volume of intestinal fluid at 200 and 400 mg/kg doses as compared to the negative control. On the contrary, the 80% methanolic extract at 100 mg/kg was devoid of significant inhibition on both parameters. The result revealed that the effect of the test extract at 400 mg/kg on the weight of the intestinal content was comparable with that of the standard drug (51.3%). The findings in this model may indicate that the plant extract has a significant antisecretory effect, and this contributes to its antidiarrheal effect noted in the castor oil-induced diarrhea model. The active metabolite of castor oil ricinoleic acid is responsible for releasing prostaglandins and, thus, leading over intestinal secretion by preventing the reabsorption of sodium chloride and water [50]. Thus, it is possible to infer that the extract significantly inhibits gastrointestinal hypersecretion and enteropooling by increasing reabsorption of electrolytes and water. Studies reported that the active metabolite ricinoleic acid might activate the nitric oxide pathway and induce nitric oxide (NO) dependent gut secretion [26]. Other studies also confirmed that NO is involved in the causation of diarrhea, and this is counteracted by agents that inhibit NO synthesis [51]. Therefore, the reduction of the concentration of nitric oxide in the small intestine by the test extract may also be another possible mechanism by which the extract exhibits its antidiarrheal effect.

The antienteropooling activity of the extract could also probably be related to the existence of phytochemical constituents including flavonoids and tannins. Flavonoids inhibit the release of prostaglandins, thereby inhibiting secretion induced by castor oil and facilitating absorption of electrolytes [43]. Tannins decrease fluid secretion by inhibiting CFTR and CaCC, by generating a protein-precipitating reaction on the GI mucosa [44, 52].

Generally, the ADI value indicates a measure of how much effective an extract is in treating diarrhea [26]. The ADI value increased with dose, suggesting the dose-dependency nature of the parameter. The highest selected dose of the extract, with the highest ADI value, is endowed with better antidiarrheal activity when compared with other selected doses.

According to previous studies, the plant extract has excellent in vitro antibacterial activities against diarrhegenic pathogens [20]. Therefore, in addition to its antimotility and antisecretory activities, *Cordia africana* can also be effective for treating diarrhea due to bacterial pathogens.

## 5. Conclusions

The findings of the present study demonstrated that the hydromethanolic extract of the leaves of *Cordia africana* is endowed with a promising antidiarrheal activity. The exact mechanism of action of the crude extract for its antidiarrheal activities is not known, but the antisecretory and antimotility activities might be the possible mechanisms by which the leaf extract could render this beneficial effect. The aforementioned activities may be attributedto the presence of active phytochemical constituents including flavonoids, tannins, terpenoids, saponins, phenols, and alkaloids that act either individually or collectively to bring about the overall antidiarrheal effect. Hence, the present work validates the use of *Cordia africana* leaf for treating diarrheal disease in Ethiopian folklore medicine.

## Figures and Tables

**Table 1:** Preliminary phytochemical screening of hydromethanolic extract of *C. africana* leaf.

Phytochemical constituents	Methods used for screening	Result
Flavonoids	Lead acetate test	Positive
Terpenoids	Salkowski test	Positive
Glycosides	Glycoside test	Negative
Alkaloids	Wagner's test	Positive
Phenols	NaOH test	Positive
Tannins	Ferric chloride test	Positive
Saponins	Foam test	Positive
Steroids	Salkowski's test	Negative

Note. Positive: present; negative: absent.

**Table 2:** Effect of crude extract of the leaves of *Cordia africana* on castor oil-induced diarrhea in mice.

Dose (mg/kg)	Onset of diarrhea (min)	No. of wet feces	Tot. no. of feces	Wt. of wet feces (gm)	Wt. of total feces (gm)	% Inhi. of defecation	%WWFO	%WTFO
Control	49.8 ± 3.75	7.3 ± 0.72	10.8 ± 0.70	1.36 ± 0.04	1.51 ± 0.05	—	—	—
80ME 100	81.2 ± 2.09^b^^*∗*^	6.5 ± 0.43	9.2 ± 0.48	1.10 ± 0.06^b^^*∗*^	1.13 ± 0.16^b^^*∗*^	11	80.9	74.8
80ME 200	96.5 ± 2.49^b^^*∗∗*^	1.7 ± 0.33^b^^*∗∗*^	3.3 ± 0.42^b^^*∗∗*^	0.29 ± 0.04^b^^*∗∗*^	0.41 ± 0.04^b^^*∗∗*^	76.7	21.3	27.2
80ME 400	109.7 ± 5.29^b^^*∗∗*^	1.2 ± 0.40^b^^*∗∗*^	2.7 ± 0.21^b^^*∗∗*^	0.16 ± 0.05^b^^*∗∗*^	0.25 ± 0.04^b^^*∗∗*^	83.6	11.8	16.6
Loperamide 3	128.3 ± 9.10^b^^*∗∗*^	0.7 ± 0.33^b^^*∗∗*^	2.0 ± 0.37^b^^*∗∗*^	0.10 ± 0.04^b^^*∗∗*^	0.18 ± 0.05^b^^*∗∗*^	90.4	7.4	11.9

Values are expressed as mean ± standard error of the mean (*n* = 6); analysis was done using one-way analysis of variance followed by Tukey post hoc test; ^b^compared with negative control values. wt. = weight; ME = methanol extract; min = minute; gm = gram; WWFO = weight of wet fecal output; WTFO = weight of total fecal output; Inhi. = inhibition. ^*∗*^*P* < 0.01; ^*∗∗*^*P* < 0.001.

**Table 3:** Effect of crude extract of the leaves of *Cordia africana* on gastrointestinal transit in mice.

Dose (mg/kg)	Length of the small intestine (cm)	Distance travelled by charcoal meal (cm)	Peristaltic index (%)	% Inhibition
Control	51 ± 0.97	44.5 ± 0.76	87.3 ± 1.51	—
80ME 100	52.2 ± 1.08	42.7 ± 0.95	83.7 ± 1.18	4.1
80ME 200	50.2 ± 1.08	23.3 ± 0.49^b^^*∗*^	46.6 ± 1.38^b^^*∗*^	47.6
80ME 400	48.5 ± 1.69	13.0 ± 0.97^b^^*∗*^	26.6 ± 1.23^b^^*∗*^	70.8
Loperamide 3	51.8 ± 1.30	12.7 ± 0.72^b^^*∗*^	24.5 ± 1.57^b^^*∗*^	71.5

Values are expressed as mean ± standard error of the mean (*n* = 6); analysis was done using one-way analysis of variance followed by Tukey post hoc test. ^b^Compared with negative control values. ME = methanol extract; cm = centimeter. ^*∗*^*P* < 0.001.

**Table 4:** Effect of crude extract of the leaves of *Cordia africana* on intestinal fluid accumulation in mice.

Dose (mg/kg)	Volume of intestinal content (ml)	% Inhibition	Weight of intestinal content (gm)	% Inhibition
Control	1.30 ± 0.10	—	1.50 ± 0.09	—
80ME 100	1.16 ± 0.11	10.8	1.17 ± 0.07	22
80ME 200	0.75 ± 0.08^b^^*∗*^	42.3	0.85 ± 0.08^b^^*∗∗*^	43.3
80ME 400	0.50 ± 0.10^b^^*∗∗*^	61.5	0.73 ± 0.15^b^^*∗∗*^	51.3
Loperamide 3	0.42 ± 0.06^b^^*∗∗*^	67.7	0.70 ± 0.06^b^^*∗∗*^	53.3

Values are expressed as mean ± standard error of the mean (*n* = 6); analysis was done using one-way analysis of variance followed by Tukey post hoc test. ^b^Compared with negative control values. ME = methanol extract; ml = millimeter; gm = gram. ^*∗*^*P* < 0.01; ^*∗∗*^*P* < 0.001.

**Table 5:** In vivo antidiarrheal indices of crude extract of the leaves of *Cordia africana* in mice.

Dose (mg/kg)	Dfreq	Gmeq	Pfreq	ADI
Control	—	—	—	—
80ME 100	63.1	4.1	11	12.2
80ME 200	93.8	47.6	76.7	69.9
80ME 400	122.2	70.8	83.6	89.8
Loperamide 3	157.6	71.5	90.4	100.6

ME = methanol extract; Dfreq = delay in diarrheal onset; Gmeq = gut meal travel reduction; Pfreq = purging frequency; ADI = antidiarrheal index.

## Data Availability

The datasets analyzed during the current study are available from the corresponding author upon reasonable request.

## Abbreviations

CA: *Cordia africana*
ADI: Antidiarrheal index
CaCC: Calcium-activated chloride channel
CFTR: Cystic fibrosis transmembrane conductance regulator
CO: Castor oil
COX: Cyclooxygenase
GI: Gastrointestinal
HT: Hydrotyramine.

## References

[1] Teferi M. Y., Abdulwuhab M., Yesuf J. S. (2019). Evaluation of in vivo antidiarrheal activity of 80% methanolic leaf extract of Osyris quadripartita Decne (Santalaceae) in Swiss Albino Mice. *Journal of evidence-based integrative medicine*.

[2] Manetu W. M., M’masi S., Recha C. W. (2021). Diarrhea disease among children under 5 years of age: a global systematic review. *Open Journal of Epidemiology*.

[3] Tadesse E., Engidawork E., Nedi T., Mengistu G. (2017). Evaluation of the anti-diarrheal activity of the aqueous stem extract of Lantana camara Linn (Verbenaceae) in mice. *BMC Complementary and Alternative Medicine*.

[4] Troeger C., Khalil I. A., Rao P. C. (2018). Rotavirus vaccination and the global burden of rotavirus diarrhea among children younger than 5 years. *JAMA Pediatrics*.

[5] Kim H. S. (2005). Do not put too much value on conventional medicines. *Journal of Ethnopharmacology*.

[6] Pathak K., Das R. J. (2013). Herbal medicine-a rational approach in health care system. *International Journal of Herbal Medicine*.

[7] Tiwari S. (2008). Plants: a rich source of herbal medicine. *Journal of Natural Products*.

[8] d’Avigdor E., Wohlmuth H., Asfaw Z., Awas T. (2014). The current status of knowledge of herbal medicine and medicinal plants in Fiche, Ethiopia. *Journal of Ethnobiology and Ethnomedicine*.

[9] Enyew A., Asfaw Z., Kelbessa E., Nagappan R. (2013). Status of medico-cultural commercial plants at Fiche town market, Ethiopia. *International Journal of Pharmaceuticals and Health Care Research*.

[10] Etana B. (2010). *Ethnobotanical Study of Traditional Medicinal Plants of Goma Wereda, Jima Zone of Oromia Region*.

[11] Tadesse B., Mulugeta G., Fikadu G., Sultan A., Nekemte E. (2014). Survey on ethno-veterinary medicinal plants in selected Woredas of east Wollega zone, western Ethiopia. *Journal of Biology, Agriculture and Healthcare*.

[12] Alemayehu G., Asfaw Z., Kelbessa E. (2016). Cordia africana (Boraginaceae) in Ethiopia: a review of its taxonomy, distribution, ethnobotany and conservation status. *International Journal of Botany Studies*.

[13] Wondafrash D. Z., Bhoumik D., Altaye B. M., Tareke H. B., Assefa B. T. (2019). Antimalarial activity of Cordia africana (Lam.) (Boraginaceae) leaf extracts and solvent fractions in plasmodium berghei-infected mice. *Evidence-Based Complementary and Alternative Medicine*.

[14] Woldeab B., Regassa R., Alemu T., Megersa M. (2018). Medicinal plants used for treatment of diarrhoeal related diseases in Ethiopia. *Evidence-Based Complementary and Alternative Medicine: eCAM*.

[15] Asrie A. B., Abdelwuhab M., Shewamene Z., Gelayee D. A., Adinew G. M., Birru E. (2016). Antidiarrheal activity of methanolic extract of the root bark of Cordia africana. *Journal of Experimental Pharmacology*.

[16] Teklay A., Abera B., Giday M. (2013). An ethnobotanical study of medicinal plants used in Kilte Awulaelo District, Tigray Region of Ethiopia. *Journal of Ethnobiology and Ethnomedicine*.

[17] Tijjani R., Zezi A., Shafiu R., Umar M. (2015). Anti-inflammatory and antioxidants properties of the ethanolic stem bark extract of Cordia africana (Lam.). *Annals of Phytomedicine*.

[18] Alhadi E. A., Khalid H. S., Alhassan M. S. (2015). Antioxidant and cytotoxicity activity of Cordia africana in Sudan. *Advancement in Medicinal Plant Research*.

[19] Tijjani R., Umar M., Hussaini I., Shafiu R., Zezi A. (2016). Antinociceptive activities of the ethanolic stem bark extract of Cordia africana (Boraginaceae) in rats and mice. *Annals of Biological Sciences*.

[20] Isa A. I., Saleh M. I. A., Abubakar A. (2016). Evaluation of anti-inflammatory, antibacterial and cytotoxic activities of Cordia africana leaf and stem bark extracts. *Bayero Journal of Pure and Applied Sciences*.

[21] Mekonnen B., Asrie A. B., Wubneh Z. B. (2018). Antidiarrheal activity of 80% methanolic leaf extract of Justicia schimperiana. *Evidence-Based Complementary and Alternative Medicine*.

[22] Alam S., Bhatnagar S. (2006). Current status of anti-diarrheal and anti-secretory drugs in the management of acute childhood diarrhea. *The Indian Journal of Pediatrics*.

[23] National Research Council (1985). *Guide for the Care and Use of Laboratory Animals*.

[24] Geetha T., Geetha N. (2014). Phytochemical screening, quantitative analysis of primary and secondary metabolites of Cymbopogan citratus (DC) Stapf. leaves from Kodaikanal hills, Tamilnadu. *International Journal of pharmtech research*.

[25] OECD (2008). TN. 425: acute oral toxicity: up-and-down procedure. *OECD Guidelines for the Testing of Chemicals*.

[26] Sisay M., Engidawork E., Shibeshi W. (2017). Evaluation of the antidiarrheal activity of the leaf extracts of Myrtus communis Linn (Myrtaceae) in mice model. *BMC Complementary and Alternative Medicine*.

[27] Abdela J. (2019). Evaluation of in vivo antidiarrheal activities of hydroalcoholic leaf extract of dodonaea viscosa L. (Sapindaceae) in Swiss albino mice. *Journal of Evidence-Based Integrative Medicine*.

[28] Than A., Kulkarni H. J., Hmone W., Tha S. J. (1989). Anti-diarrhoealefficacy of some Burmese indigenous drug formulations in experimental diarrhoeal test models. *International Journal of Crude Drug Research*.

[29] Adeyemi O. O., Akindele A. J. (2008). Antidiarrhoeal activity of the ethyl acetate extract of Baphia nitida (Papilionaceae). *Journal of Ethnopharmacology*.

[30] Robert A., Nezamis J. E., Lancaster C., Hanchar A. J., Klepper M. S. (1976). Enteropooling assay: a test for diarrhea produced by prostaglandins. *Prostaglandins*.

[31] Palombo E. A. (2006). Phytochemicals from traditional medicinal plants used in the treatment of diarrhoea: modes of action and effects on intestinal function. *Phytotherapy Research*.

[32] Horton E. W., Main I. H., Thompson C. J., Wright P. M. (1968). Effect of orally administered prostaglandin E1 on gastric secretion and gastrointestinal motility in man. *Gut*.

[33] Imam M. Z., Sultana S., Akter S. (2012). Antinociceptive, antidiarrheal, and neuropharmacological activities ofBarringtonia acutangula. *Pharmaceutical Biology*.

[34] Capasso F., Mascolo N., Izzo A. A., Gaginella T. S. (1994). Dissociation of castor oil-induced diarrhoea and intestinal mucosal injury in rat: effect of NG-nitro-L-arginine methyl ester. *British Journal of Pharmacology*.

[35] Uchida M., Kato Y., Matsueda K., Shoda R., Muraoka A., Yamato S. (2000). Involvement of nitric oxide from nerves on diarrhea induced by castor oil in rats. *The Japanese Journal of Pharmacology*.

[36] Salgado H. R. N., Roncari A. F. F., Moreira R. R. D. (2005). Antidiarrhoeal effects of Mikania glomerata Spreng. (Asteraceae) leaf extract in mice. *Revista Brasileira de Farmacognosia*.

[37] Fischbach W., Andresen V., Eberlin M., Mueck T., Layer P. (2016). A comprehensive comparison of the efficacy and tolerability of racecadotril with other treatments of acute diarrhea in adults. *Frontiers of Medicine*.

[38] Molla M., Gemeda N., Abay S. A. (2017). Investigating potential modes of actions of Mimusops kummel Fruit extract and solvent fractions for their antidiarrheal activities in mice. *Evidence-Based Complementary and Alternative Medicine*.

[39] Nu R., Aslam K., Urooj F. (2013). Presence of laxative and antidiarrheal activities in Periploca aphylla: a Saudi medicinal plant. *International Journal of Pharmacology*.

[40] Awouters F., Niemegeers C. J., Lenaerts F. M., Janssen P. A. (1978). Delay of castor oil diarrhoea in rats: a new way to evaluate inhibitors of prostaglandin biosynthesis. *Journal of Pharmacy and Pharmacology*.

[41] Bajad S., Bedi K. L., Singla A. K., Johri R. K. (2001). Antidiarrhoeal activity of piperine in mice. *Planta Medica*.

[42] Tangpu V., Yadav A. K. (2004). Antidiarrhoeal activity of Rhus javanica ripen fruit extract in albino mice. *Fitoterapia*.

[43] Hämäläinen M., Nieminen R., Asmawi M. Z., Vuorela P., Vapaatalo H., Moilanen E. (2011). Effects of flavonoids on prostaglandin E2 production and on COX-2 and mPGES-1 expressions in activated macrophages. *Planta Medica*.

[44] Ashok P. K., Upadhyaya K. (2012). Tannins are astringent. *Journal of Pharmacognosy and Phytochemistry*.

[45] Pérez-Gutiérrez S., Zavala-Mendoza D., Hernández-Munive A., Mendoza-Martínez Á, Pérez-González C., Sánchez-Mendoza E. (2013). Antidiarrheal activity of 19-deoxyicetexone isolated from Salvia ballotiflora Benth in mice and rats. *Molecules*.

[46] Hansen M. B. (2003). Neurohumoral control of gastrointestinal motility. *Physiological Research*.

[47] Chen W., Chung H.-H., Cheng J.-T. (2012). Opiate-induced constipation related to activation of small intestine opioid *μ*2-receptors. *World Journal of Gastroenterology*.

[48] Di Carlo G., Autore G., Izzo A. (1993). Inhibition of intestinal motility and secretion by flavonoids in mice and rats: structure-activity relationships. *Journal of Pharmacy and Pharmacology*.

[49] Channa S., Dar A., Ahmed S., Atta-ur-Rahman (2005). Evaluation of Alstonia scholaris leaves for broncho-vasodilatory activity. *Journal of Ethnopharmacology*.

[50] Pierce N. F., Carpenter C. C. J., Elliott H. L., Greenough W. B. (1971). Effects of prostaglandins, theophylline, and cholera exotoxin upon transmucosal water and electrolyte movement in the canine jejunum. *Gastroenterology*.

[51] Izzo A. A., Gaginella T. S., Mascolo N., Borrelli F., Capasso F. (1996). NG-nitro-L-arginine methyl ester reduces senna- and cascara-induced diarrhoea and fluid secretion in the rat. *European Journal of Pharmacology*.

[52] Wongsamitkul N., Sirianant L., Muanprasat C., Chatsudthipong V. (2010). A plant-derived hydrolysable tannin inhibits CFTR chloride channel: a potential treatment of diarrhea. *Pharmaceutical Research*.

